# The single-grain method: adding TEM to the equation

**DOI:** 10.1080/00173134.2019.1666915

**Published:** 2019-10-24

**Authors:** Silvia Ulrich, Friđgeir Grímsson

**Affiliations:** Department of Botany and Biodiversity Research, University of Vienna, Vienna, Austria

**Keywords:** fossil pollen, light microscopy, pollen morphology, pollen ultrastructure, scanning electron microscopy, transmission electron microscopy

## Abstract

An advanced protocol to prepare single extant and fossil pollen grains for transmission electron microscopy (TEM) analysis allows for the fast recovery of data on the ultrastructure of pollen/spores. The protocol is easy to apply and less time consuming than previous methods. The ‘loss’ of pollen grains and pollen that is ‘difficult to locate’ within the embedding material is avoided, and each single pollen grain can be prepared successfully for TEM analysis. This preparation method is meant as an addition to the single-grain method using combined light and scanning electron microscopy to investigate dispersed fossil pollen grains developed by Dr Reinhard Zetter in the late 1980s.

The best way to identify fossil pollen grains is to compare them with pollen from extant plants. In most palaeobotanical literature, such a comparison is based on morphological features that can be observed using light microscopy (LM) only. This is despite the fact that over the past 50 years papers documenting morphology and ultrastructure in pollen of extant plants have accumulated drastically. It is now considered a standard to present modern pollen simultaneously using LM, scanning electron microscopy (SEM) and transmission electron microscopy (TEM).

Already in the early 1970s, Sivak (1973, pp. 398–399, in French) proposed a combined method to investigate single fossil pollen grains with both LM and SEM. His method applied micropipettes to transport pollen from the original sample (organic residue) into a fresh drop of glycerine on a microscope slide. The pollen was then covered with an additional slide and the grain could be photographed under LM from two sides (by turning the slide). This method is unpractical since it does not allow for the free manipulation of the pollen grain observed in LM, and the grain can only be studied from one view. Also, the constant transport using micropipettes during washing and for final placement on the SEM stub increases the risk of losing the pollen grain. In the mid-1970s, Doyle et al. (1975, pp. 443–444) applied a modified version of Sivak’s method to study single fossil pollen grains with both LM and SEM. They also studied single fossil grains with both LM and TEM. For the TEM preparations, pollen grains were moved (following LM photography) with a micropipette into a thin layer of agar on a microscope slide or in a shallow petri dish. The sample was then heated (dried) to seal the pollen inside the agar. A small block containing the pollen was then cut out from the agar and stained using potassium permanganate (KMnO_4_) in distilled water. The specimen block was then washed, dehydrated using an alcohol–popylene oxide series, and embedded in epon. The block was then sectioned using a diamond knife, and the sections stained with uranyl acetate and lead citrate. A few years later, Daghlian (1982, pp. 540–543) presented a ‘new’ single-grain method, combining LM, SEM and TEM, applicable for fossil pollen. Even though, in the title of his paper, it is stated as a ‘simple’ method, that is not really the case. Following preparation of the sediment the organic residue was mounted on a microscope slide in Flo-texx plastic without a cover glass. The grains of interest were marked in the Flo-texx with a gauge hypodermic needle and their coordinates recorded. The microscope slide was then covered with a cover glass and immersion oil for LM photography. Following LM observations, the cover glass and immersion oil were removed with alcohol. Individually marked pollen grains were relocated on the slide using the recorded coordinates and marks in the plastic. Single pollen grains were then cut out of the plastic and transferred to filters where they were washed with toluene and dried. The pollen grains were transported along with the filter, or part of it, onto a SEM stub, using a moistened eyelash. Following SEM observations, the grains were transported and prepared for TEM according to the procedure presented by Doyle et al. (1975). In the following years, various adjustments to the single-grain method appeared, e.g. Zavada and Crepet (1985), Ward et al. (1989), Kurmann and Doyle (1994), Zavada (2007), Tekleva and Denk (2012), and Zavialova et al. (2018).

At the end of the 1980s, Zetter (1989, in German; see also Ferguson et al. 2007; Halbritter et al. 2018, pp. 119–121), presented yet a new combined method, involving an erect image compound microscope and a micromanipulator (nasal hair), to study single fossil pollen grains in both LM and SEM. This was the first really fast and easy way to study single fossil pollen grains in both LM and SEM that also minimised the risk of losing the grains. The grains had only to be transported twice, and without any effort the pollen would attach to the nasal hair when soaked in glycerine. This method has been used in numerous papers by Reinhard Zetter and his colleagues since its introduction 30 years ago (see publication list of R. Zetter, in this issue of *Grana*).

Despite the great number of publications on (single) fossil pollen, TEM has rarely been included. This becomes clear when publications from the two main palynological journals are screened (Table I). In 2018, *Grana* published 29 papers, six of them on fossil pollen/spores, and one of them included TEM. In the last nine years, *Grana* has published 243 papers, 33 of them on fossil pollen/spores, and five of them including TEM. Similar scenarios are observed from the publications appearing in the journal *Palynology* (Table I). In 2018, *Palynology* published 52 papers, nine of them on fossil pollen/spores, and none of them included TEM. In the last nine years, *Palynology* has published 271 papers, 72 of them on fossil pollen/spores, and only two of them including TEM. It is difficult to find any good reason for this lack of TEM analysis, but we speculate that the time consuming process of preparing single grains for TEM, the ‘loss’ of grains during preparation, and the expenses play important roles.

**Table I.** Documentation of fossil pollen/spores (pre-Holocene) in two journals acknowledged for palaeopalynology (last nine years are shown).

	Number of papers	Papers on fossil pollen/spores	LM micrographs (only)	SEM micrographs (only)	LM and SEM micrographs	LM, SEM, and TEM micrographs
*Grana* 2018	29	6	1	1	2	1
*Grana* 2017	40	5	0	1	4	0
*Grana* 2016	17	6	2	0	4	0
*Grana* 2015	24	3	0	1	2	0
*Grana* 2014	25	4	0	3	1	0
*Grana* 2013	30	1	0	0	0	1
*Grana* 2012	28	1	0	0	0	(1)
*Grana* 2011	22	4	0	1	2	1
*Grana* 2010	28	3	0	1	1	1
Total	243	33	3	8	16	5
*Palynology* 2018	52	9	7	0	0	0
*Palynology* 2017	66	16	9	0	3	(1)
*Palynology* 2016	28	7	7	0	0	0
*Palynology* 2015	25	8	5	0	1	0
*Palynology* 2014	24	5	5	0	0	0
*Palynology* 2013	23	6	3	0	2	0
*Palynology* 2012	29	11	9	0	1	(1)
*Palynology* 2011	12	4	3	0	1	0
*Palynology* 2010	12	6	4	0	1	0
Total	271	72	52	0	9	2

Note: Numbers in parentheses show papers without LM micrographs.

Here, an advanced and fast protocol to prepare single fossil pollen grains for TEM is presented. The method is not only fast, but also non-toxic, and shortens the TEM preparation down to a day. This TEM preparation method completes the ‘user-friendly’ single-grain method developed by Reinhard Zetter in the late 1980s, and makes it possible to go from sedimentary sample to single grains photographed in LM, SEM, and TEM, in less than four working days.

## Material and methods

### Equipment and chemicals

For the preparation of samples for TEM analysis the following equipment and chemicals are needed: binocular microscope, oven, micromanipulator, section manipulators, lids of microcentrifuge tubes, pipettes, sections from plastic pipettes, forceps, razor blades, ultramicrotome, diamond knife (ultramicrotome), formvar film-coated copper grids with a slot, loop, embedding media (agar low-viscosity resin, Spurr’s low-viscosity epoxy resin), glycerine, acetone, ethanol, staining agents (KMnO_4_, toluidine blue), two-component epoxy adhesive glue or cyanoacrylate adhesive (Krazy Glue).

Most of the equipment used for the TEM preparation is expensive. There are ways to reduce the cost by producing, e.g. your own loops (Halbritter et al. 2018, p. 110), formvar film-coated grids (Halbritter et al. 2018, p. 108), and micromanipulator (Halbritter et al. 2018, pp. 110, 122). There are various embedding moulds on the market, many of which are unsuitable or expensive. Instead of using standard flat embedding moulds, made of silicon rubber, Teflon or disposable plastic moulds, the use of plastic lids from microcentrifuge tubes (this study) proved to be practical for single fossil pollen grains.

### Test specimens

The method was tested on three different specimens, each including a single pollen grain. A single dispersed, recent pollen grain from a surface soil sample collected in a forested area close to Langenzersdorf, Lower Austria, was selected for TEM after acetolysis (see Halbritter et al. 2018, p. 101). Secondly, a single dispersed fossil pollen grain extracted from an early Miocene sample from Mush, Ethiopia (see table IV in Grímsson et al. 2019) was prepared for TEM after the sedimentary sample was prepared following the protocol of Grímsson et al. (2008) and the pollen was studied in both LM and SEM as described in Zetter (1989) and Halbritter et al. (2018). Finally, a single *in situ* fossil pollen grain was extracted from the anther of a fossil flower from the middle Eocene Messel locality, Germany (e.g. Schaal & Ziegler 1988), using a micromanipulator. After ‘rapid acetolysis’ (Halbritter et al. 2018, p. 103) on a microscope slide the pollen grain was transferred into glycerine on a glass slide and investigated by LM and SEM as described in Zetter (1989) and Halbritter et al. (2018) before preparation for TEM.

## Method protocol

**Figure 1.**
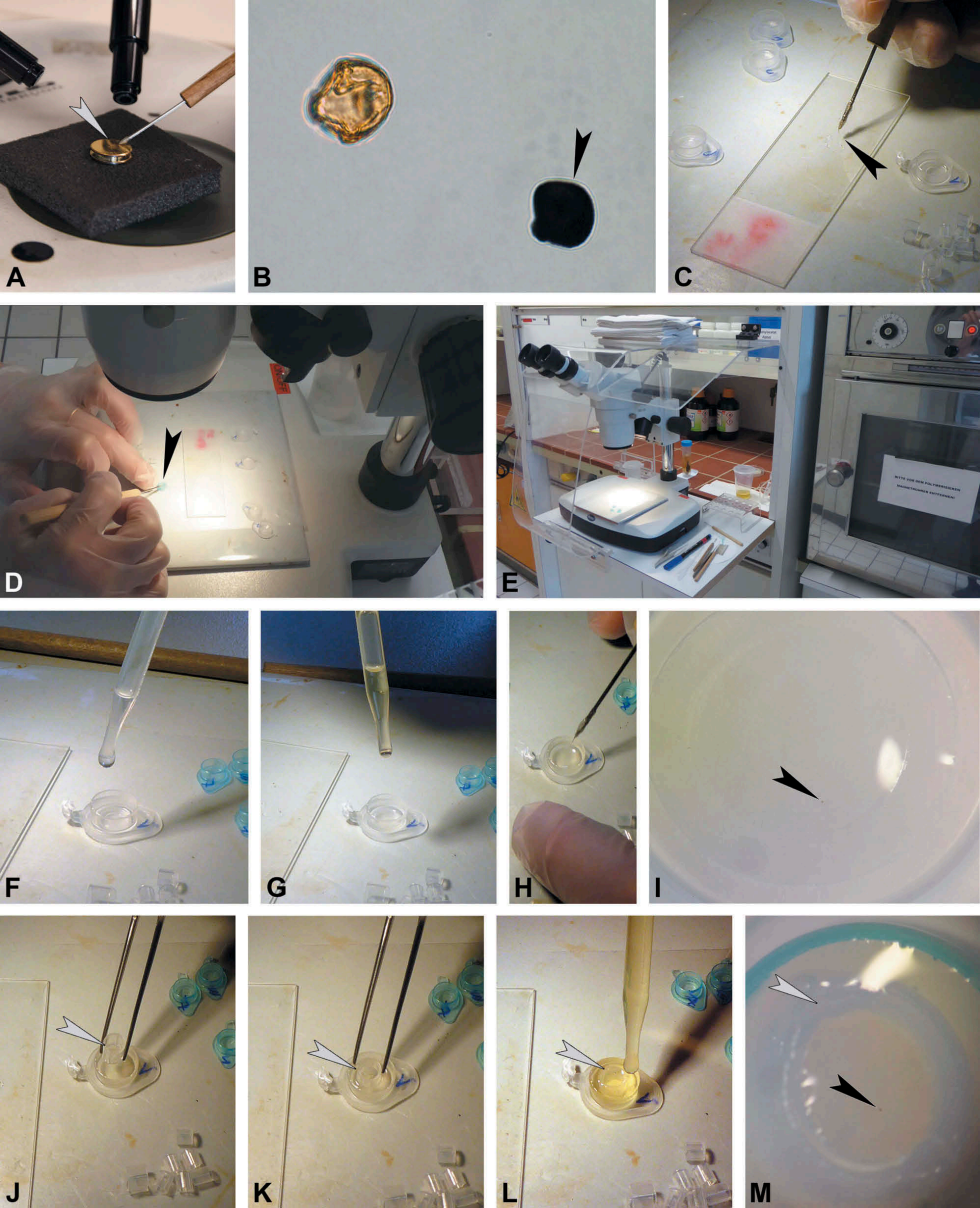
Protocol for the preparation of single fossil pollen grains for TEM, part 1 (fixation and infiltration). **A.** Fossil pollen picked-up with micromanipulator (nasal hair; *arrowhead*) from SEM stub. **B.** Two fossil pollen grains seen under LM, black grain is coated with gold (*arrowhead*). **C, D.** Pollen picked-up from glycerine with micromanipulator (**C**, *arrowhead*) and transferred into embedding mould (**D**, *arrowhead*). **E.** Equipment setup, binocular under fume hood. **F, G.** Embedding mould, a microcentrifuge tube lid, filled with acetone (**F**) and embedding media (**G**). **H, I.** Micromanipulator used to place the pollen (**I**, *arrowhead*) in the ± middle of the embedding mould. **J–L.** Placing plastic pipette section (*arrowhead*) over pollen grain, adding embedding media. **M.** Pollen grain (*black arrowhead*) positioned inside the pipette section (*white arrowhead*).

**Figure 2.**
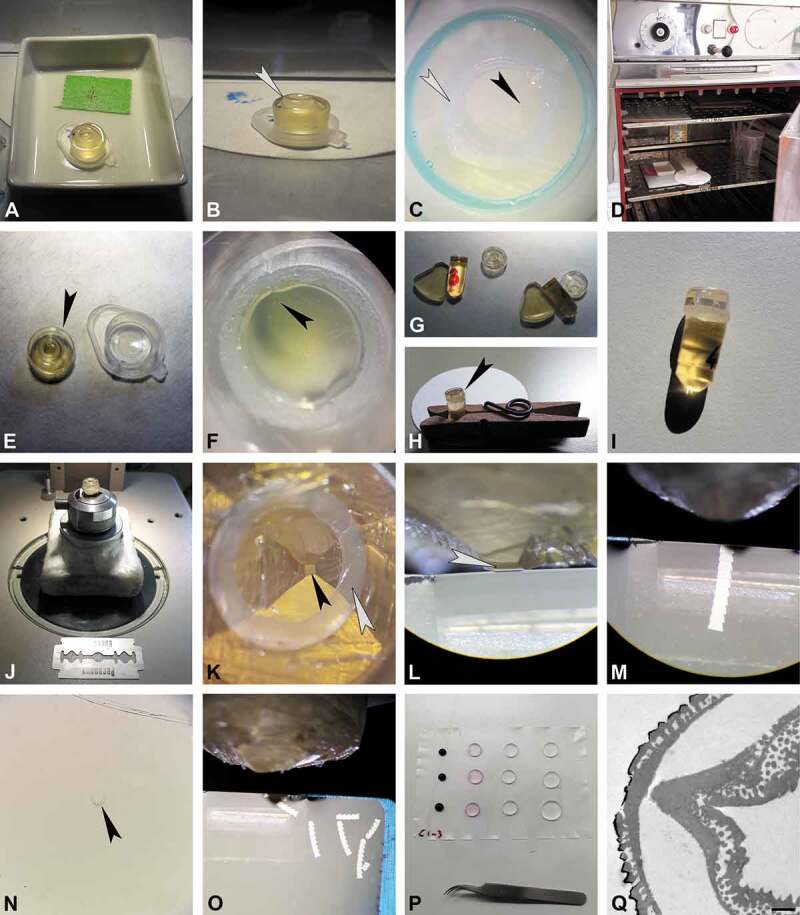
Protocol for the preparation of single fossil pollen grains for TEM, part 2 (infiltration, embedding, ultramicrotomy, and staining). **A.** Infiltration at room temperature until acetone evaporates. **B.** Embedding mould re-filled with embedding media, make sure not to overfill (*arrowhead*). **C.** Final position of pollen grain (*black arrowhead*) inside plastic pipette section (*white arrowhead*). **D.** Polymerisation of sample and block holder in oven. **E.** Polymerised specimen block (*arrowhead*) freed using a racer blade. **F.** Pollen grain (*arrowhead*) observed in polymerised specimen block under a binocular. **G–I.** Specimen block (*arrowhead*) fixed on a pre-made block, use a clip (**H**) for stability. **J.** Trimming under binocular. **K.** Trimmed block face trapezoid in form, with pollen grain at the tip (*black arrowhead*), pipette section seen as a broad white circle (*white arrowhead*). **L–M.** Ultra-thin sectioning of specimen block with diamond knife, *arrowhead* pointing to pollen grain. **N.** Single section (*arrowhead*) stained with toluidine blue observed in LM. **O.** Ready-made section sequences. **P.** Staining sections with potassium permanganate (KMnO_4_). **Q.** TEM section showing final result; KMnO_4_ staining. Scale bar – 1 µm (Q).

Following a combined LM and SEM investigation of the same pollen grain, the stub with the coated pollen is placed under a binocular (Figure 1A). The micromanipulator (nasal hair attached to a teasing needle) is dipped into glycerine and used to pick up the fossil grain from the SEM stub. When the glycerine soaked micromanipulator is brushed against the pollen, laying on the SEM stub, the grain will adhere to it and can be transferred into a drop of glycerine on a new microscope slide. Dipping the micromanipulator into the glycerine drop on the microscope slide will release the pollen from the hair, and the grain will stay in the drop when the hair is pulled out (Figure 1B). For the TEM preparation, the pollen grain is transferred from the glycerine drop directly into the embedding mould (Figure 1C, D). This step is also carried out using a micromanipulator under a microscope or binocular. Since some of the chemical used for the embedding/fixation can be harmful or toxic (see Discussion section) further use of the binocular should be conducted under a fume hood (Figure 1E). It is important to turn the hood setting on the lowest intensity (Step 1) in order not to lose the pollen grain due to strong airflow. To make it easy finding the single pollen grain, within the embedding mould, a microcentrifuge tube lid (with a flat/smooth inner surface; Figure 1F) is used for infiltration and embedding. The embedding mould must be cleaned before embedding, as particles (fibres, dust, recent pollen, etc.) might interfere with the embedding material. Acetone or ethanol is added dropwise into the mould and a pipette is used to reabsorb and dispose of the liquid (including disturbing particles). This step is repeated until the embedding mould is fully clean. The sputter coated pollen grain is then transferred from the drop of glycerine, using a micromanipulator, into the clean embedding mould (Figure 1D). Striking the micromanipulator along the bottom of the embedding mould will transfer the pollen grain into the mould where it will remain (glycerine makes it sticky). Acetone is added dropwise into the embedding mould, until the mould is half-full, in order to clean and dehydrate the pollen grain (Figure 1F). For infiltration, a few drops of fresh embedding media (1:2, acetone/LV-resin) are added dropwise into the embedding mould (Figure 1G). Agar low-viscosity resin (LV-resin; Agar Scientific, 2004) and Spurr’s low-viscosity epoxy resin (Spurr, 1969) are suitable embedding media that provide a complete and uniform penetration of the fossil pollen resulting in a satisfactory sectioning (Halbritter et al. 2018). The micromanipulator is then used to move the pollen towards the centre of the embedding mould (Figure 1H, I). To prevent the pollen grain from adhering to the wall of the embedding mould, a small section from a plastic pipette is used to restrain the pollen grain in the centre of the embedding mould. Using forceps, the pipette section is placed into the embedding mould and over the pollen grain (Figure 1J–M). The plastic section is soft and easily trimmed with a razor blade or microtome following polymerisation. During infiltration, the sample stands for two to three hours at room-temperature until the acetone has evaporated (Figure 2A). For polymerisation, the embedding mould is dropwise filled up with embedding media. The mould should not be overfilled since the plastic pipette section might start to float and the drifting pollen grain might then get lost (Figure 2B). Drifting pollen grains are moved and manipulated inside the resin, towards the centre, using the micromanipulator (Figure 2C). The pollen grain should now be positioned close to the centre of the plastic section. Samples are then polymerised at 70 °C for 6 to 12 h (Figure 2D). After polymerisation, the specimen block is cut out from the embedding mould using a razor blade (Figure 2E). Using the binocular, the pollen grain is now observable, positioned close to the flattened distal side of the block (Figure 2F). The specimen block is then fixed, using two-component epoxy adhesive glue or a cyanoacrylate adhesive (Krazy Glue), to a large and round, pre-made block (or another type of block holder). The premade blocks are made in standard embedding moulds using the remaining embedding resin (Figure 2G, H). While the ‘Krazy Glue’ adhesive polymerises within a minute, the polymerisation of the two-component adhesive can be sped up by placing the block in an oven at 40 °C for about 15 min. The ‘final’ fixed specimen block (Figure 2I) is now ready for ultrathin sectioning. If the orientation of the pollen grain needs to be adjusted, the part of the specimen block including the pollen is cut off, turned in the desired position, and attached to a specimen block holder. Trimming of the specimen block follows Halbritter et al. (2018, p. 110). During the trimming process, the block is trimmed from the sides only to form a trapezoid, between 2 and 4 mm^2^ (Figure 2J, K). Since the fossil pollen grain is already positioned at the appropriate level for sectioning (Figure 2K), semi-thin sections from the tip of the block face are not necessary. Also, as the surface of the specimen block is already flat it is now possible to directly produce ultra-thin sections using a diamond knife (ultramicrotome; Figure 2L, M). To establish when the fossil pollen grain has been reached, a single section is picked up with a loop and stained with toluidine blue (Figure 2N; Halbritter et al. 2018, p. 114). Final ultra-thin sections through the pollen grain are transferred onto formvar film-coated copper grids with a slot and treated with KMnO_4_ (Figure 2O, P; Halbritter et al. 2018, p. 118). For staining with KMnO_4_, the sections collected on copper grids are treated with 1% aqueous KMnO_4_ solution for 7 min and thoroughly washed three times for 5 min in a row of water drops (Figure 2P). Following staining, the sections are ready for TEM analysis (Figure 2Q).

## Discussion

### Outcome

**Figure 3.**
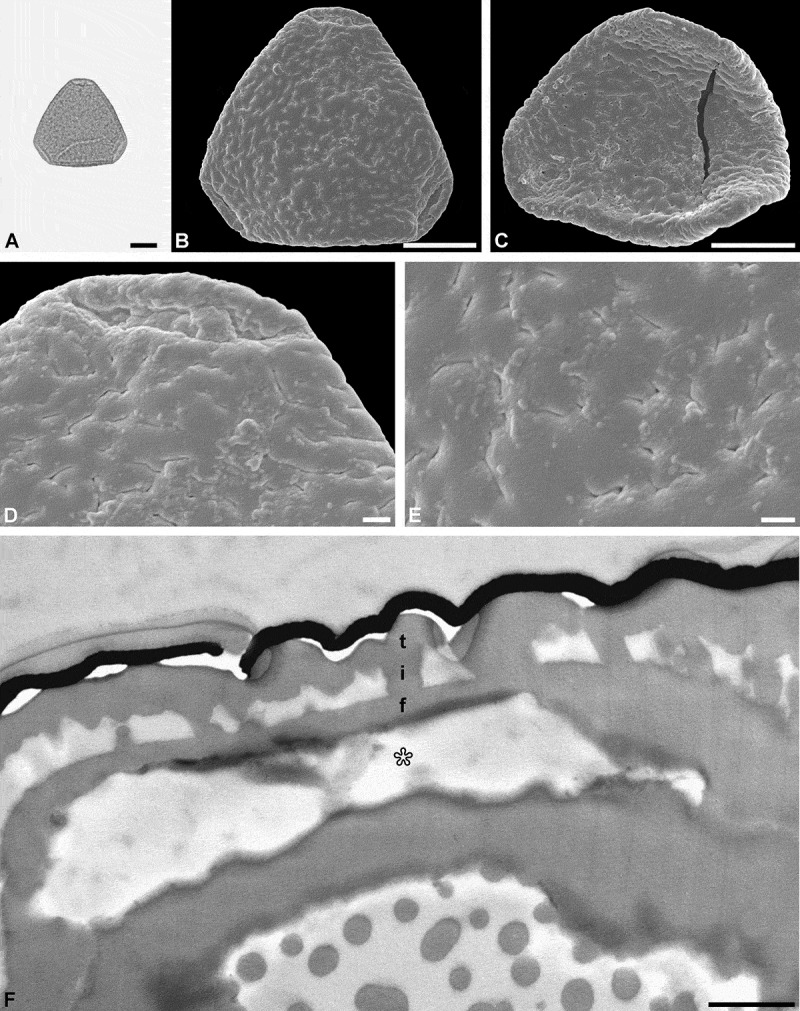
Light microscopy (LM), scanning electron microscopy (SEM) and transmission electron microscopy (TEM) micrographs of a single fossil *Sclerosperma* pollen grain from the early Miocene of Ethiopia, Africa. **A.** Pollen grain in polar view (LM), distal side. **B.** Pollen grain in polar view (SEM), distal side. **C.** Pollen grain in polar view (SEM), proximal side. **D.** Close-up of apex (SEM), showing aperture on distal side. **E.** Close-up of central polar area (SEM), distal side. **F.** Cross-section of pollen wall (TEM), unstained section (compare to Figure 4E, F), gap between pollen wall and gold layer (*black*) is formed when the formvar film is too thin. Pollen wall in aperture region (*asterisk*), tectum: eutectate (t), infratectum: columellate (i), foot layer: continuous-compact (f). Scale bars – 10 µm (A–C), 1 µm (D, E), 1 µm (F).

The combined LM, SEM and TEM micrographs of a single pollen grain complement each other and provide ‘all’ available data on pollen morphology and ultrastructure. One example shown here (test specimen 2), Figure 3, with micrographs from all three microscopes, is a fossil *Sclerosperma* pollen (e.g. Grímsson et al. 2019a, 2019b) from the early Miocene of Ethiopia (Africa). The micrographs comprising this figure were done over a course of four days. Preparing the sedimentary rock sample in the laboratory following Grímsson et al. (2008) and Halbritter et al. (2018, pp. 119–121), that included boiling the sample in hydrofluoric acid (HF), allowed for the organic residue to be isolated and studied on a microscopic slide within a single working day. The single fossil pollen grain was then studied and photographed under LM (Figure 3A), transferred onto a SEM stub, sputter coated with gold and studied and photographed using SEM (Figure 3B, D, E), following the single-grain method described by Zetter (1989) and Halbritter et al. (2018, pp. 122–123). The stub was then placed under a binocular and a drop of ethanol was added to the sputtered sample and the fossil pollen grain was turned (flipped) over, re-sputtered with gold, and again photographed using SEM (Figure 3C). The combined LM and SEM work was conducted within a single day. Following this, TEM preparation as described herein lasted a whole day. TEM micrographs (Figure 3F) were produced on the fourth working day. Applying the preparation methods described by Grímsson et al. (2008; sedimentary rock to palynomorph), Zetter (1989; combined LM and SEM analyses) and Halbritter et al. (2018; combined LM and SEM analyses) in combination with the protocol described herein (TEM preparation) allows for single fossil pollen to be displayed using LM, SEM and TEM in only four days.

### Comparing methods and results

Since the first study on the ultrastructure of extant pollen various methods have been applied to prepare pollen for TEM analysis. The most recent adjustment for preparing living pollen is by Halbritter et al. (2018), based on a classical treatment summarised by Hayat (2000). The preparation of pollen for TEM is divided into four main steps, (1) Fixation, (2) Embedding, (3) Ultramicrotomy, and (4) Staining (Table II).

**Table II.** Comparison between the different TEM preparation methods used for fossil pollen.

	Hayat 2000, Halbritter et al. 2018*	Rowley 1959	Doyle et al. 1975	Daghlian 1982	Osborn et al. 1991	Zetter et al. 2002	Zavialova et al. 2018**	This study
**Fixation**								
Prefixation	GA (6 h, room temperature)	Formalin-agar mixture concentrated into a pellet (2–3 h)	Agar-pollen pellets (min 1 h)	N/A	Pipetting grains on cellulose filters, coating both sides of filter in agar (min 1 h)	N/A	N/A	N/A
Rinsing	Buffer and distilled water (± 15 min)	Buffer and distilled water (± 15 min)	N/A	N/A	N/A	N/A	N/A	N/A
Postfixation/contrast	OsO_4_ (8–12 h, 6 °C)	OsO_4_ (45 min, 6 °C)	1% KMnO_4_ (? h)	N/A	N/A	N/A	OsO_4_ (on a cavity slide; 1–24 h)	N/A
Rinsing	Distilled water (± 15 min)	Distilled water (± 15 min)	Distilled water (± 15 min)	N/A	N/A	Ethanol	Ethanol or acetone	N/A
Dehydration	DMP (30 min) and pure acetone (30 min)	Ethanol (up to 3 h) and pure acetone	Alcohol-propylene oxide (2–3 h)	N/A	Graded ethanol series, pure acetone (with four changes to remove filter) (2–3 h)	DMP (30 min) and pure acetone (30 min)	N/A	Pollen placed in final embedding form (test tube lid), dehydration and cleaning in acetone (1 min)
Infiltration	Embedding media, dropwise ca every 6th hour (36 h)	N/A	N/A	N/A	Gradually infiltrated with embedding media (? h)	Embedding media, dropwise (8 drops) two times (8 h)	Embedding media in capsule (up to 24 h)	Embedding media, few dropwise until full (2 min)
Extraction	Acetone resin mixture removed (1 min)	N/A	N/A	N/A	?	Acetone resin mixture removed (1 min)	N/A	N/A
Evaporation	Acetone (2–3 h)	N/A	N/A	N/A	?	Acetone (2–3 h)	N/A	Acetone (2 h)
**Embedding**								
Final embedding	Osmified material cut, transferred into ‘standard’ embedding form (30 min)	Material transferred into ‘standard’ embedding form (30 min)	Material transferred into ‘standard’ embedding form (30 min)	Pollen placed in depression slide with a drop of embedding media. Drying/hardening (4–6 h). Cleaning with acetone and re-embedded into ‘standard’ embedding form (30 min)	Final embedding in shallow pans	‘flat embedding’ on glass slides covered with mould	Correct position of pollen in capsule	N/A
Polymerisation	Drying/hardening in oven (12 h)	Drying/hardening in oven (min 5 h)	Drying/hardening in oven (? h)	Drying/hardening (4–6 h)	Drying/hardening in oven (? h)	Drying/hardening in oven (12 h)	Drying/hardening in oven (min 48 h). Finding object and cutting out with scissors, polymerising again (min 48 h)	Drying/hardening in oven (6–12 h)
**Ultramicrotomy**								
Trimming	Cutting specimen block with a racer blade, forming a trapezoid (± 10 min)	?	Cutting specimen block with a racer blade, forming a trapezoid (± 10 min)	Cutting specimen block with a racer blade, forming a trapezoid (± 10 min)	?	Specimen block glued onto block (30 min), Cutting specimen block with a racer blade, forming a trapezoid (± 10 min)	Trimming second generation polymerised blocks.Cutting specimen block with a racer blade or ultramicrotome, forming a trapezoid (± 10 min)	Specimen block glued onto block (1–30 min), Cutting specimen block with a racer blade, forming a trapezoid (± 10 min)
Semi-thin sectioning	Cutting with glass knives, plain surfaced block-face, quality check of fixation, locating area for ultra-thin sectioning (30 min)	N/A	?	?	N/A	Cutting with glass knives, plain surfaced block-face, quality check of fixation, locating area for ultra-thin sectioning (30 min)	Cutting with glass knives, plain surfaced block-face, quality check of fixation, locating area for ultra-thin sectioning (30 min)	N/A
Ultra-thin sectioning	Cutting sections for TEM, sections 60–90 nm thin (1–3 h)	Cutting sections with glass knives (1–3)	Cutting sections with a diamond knife (1–3 h)	Cutting sections with a diamond knife (1–3 h)	Cutting sections with a diamond knife (1–3 h)	Cutting sections for TEM, sections 60–90 nm thin (1–3 h)	Cutting sections for TEM, sections 60–90 nm thin (1–3 h)	Cutting sections for TEM, sections 60–90 nm thin (1–3 h)
**Staining**								
U + Pb	Uranyl acetate staining, washing, lead citrate staining, washing, general contrast (1–1.5 h)	N/A	Uranyl acetate staining, washing, lead citrate staining, washing, general contrast (1–1.5 h)	Uranyl acetate staining, washing, lead citrate staining, washing, general contrast (1–1.5 h)	N/A	Uranyl acetate staining, washing, lead citrate staining, washing, general contrast (1–1.5 h)	Uranyl acetate staining, washing, lead citrate staining, washing, general contrast (1–1.5 h)	N/A
KMnO_4_ (Weber & Ulrich 2010)	N/A	N/A	N/A	N/A	N/A	N/A	N/A	KMnO_4_ staining, washing, detection of endexine (20 min)
KMnO_4_ and U + Pb (Hayat 2000)	N/A	N/A	N/A	N/A	KMnO_4_ staining, washing in water and ethanol, uranyl acetate staining, washing, lead citrate staining, washing, general contrast (1.5–2 h)	N/A	N/A	N/A
PA + TCH + SP (Weber & Frosch 1995)	N/A	N/A	N/A	N/A	N/A	Periodic acid (PA), washing, thiocarbohydrazide (TCH) washing, silver proteinate (SP) washing, general contrast (1–1.5 h)	N/A	N/A
Duration	± Five days	± Two days	± Two days	± Two days	± Two to three days	± Four days	± Four to six days	± One day

Note: (*) Standard protocol for fixation, embedding and staining living pollen; (N/A) Not applicable; (**) Many different preparation methods are described in Zavialova et al. (2018), the one listed here is the standard protocol including osmium fixation; (GA) Glutaraldehyde; (DMP) Dimethoxypropane.

Prefixation with glutaraldehyde is important to preserve the structure of the living protoplast (cell contain) in pollen, but since fossil pollen grains are without cell content there is no need for this preparation step (see Rowley 1959 versus recent studies; Table II). Some methods also apply pollen-agar pellets or pollen-filter agar pellets to entrap and/or fixate single pollen grains, reducing the chances of losing the grains (see Rowley 1959; Doyle et al. 1975; Osborn et al. 1991 versus this study; Table II). The pellets are then used for postfixation, dehydration, infiltration, and final embedding. The use of pollen-agar pellets and pollen-filter agar pellets was also tested, but proved unnecessarily time consuming and is not recommended for single fossil pollen grains.

**Figure 4.**
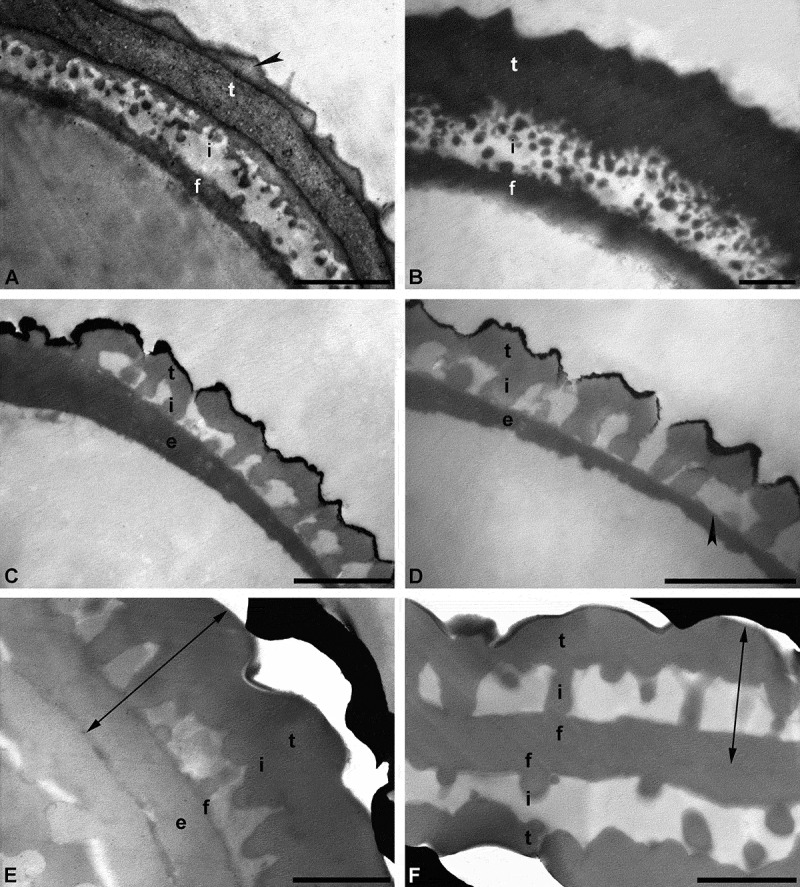
Cross-sections of pollen walls showing the effect of using different TEM staining agents. **A, B.** Pollen wall of a recent dispersed pollen grain, stained with potassium permanganate (KMnO_4_) (**A**) and uranyl acetate/lead citrate (**B**), pollen without gold coating. Pollen wall in interapertural region with tectum: eutectate (t) with inner channelled layer and outer thin layer (*arrowhead*), infratectum: granular (i), foot layer: continuous-compact (f). **C, D.** Pollen wall of a single *in situ* pollen out of a fossil flower (anther) from the Eocene of Messel, Germany, stained with KMnO_4_ (**C**) and uranyl acetate/lead citrate (**D**). Pollen wall with tectum: eutectate (t), infratectum: columellate (i), foot layer: thin and discontinuous (*arrowhead*), endexine: continuous-compact (e), outermost black layer is the gold coating. **E, F.** Pollen wall of a single dispersed fossil pollen grain from the early Miocene of Ethiopia, Africa, stained with KMnO_4_ (**E**) and uranyl acetate/lead citrate (**F**), gap between pollen wall and gold layer (*black*) is formed when the formvar film is too thin. *Two-headed arrows* indicate the exine. Pollen wall in interapertural area with tectum: eutectate (t), infratectum: columellate (i). It is uncertain if the foot layer (f) is thick (**F**) or thin (**E**), in case of the latter accompanied by a thick endexine (e). Scale bars – 1 µm (A–F).

Postfixation with osmium tetroxide is usually applied in an effort to chemically stabilise lipids and proteins, and to enhance the general contrast of cellular structure and layering of the pollen wall. Since fossil pollen are without protoplast, there is no need to use a highly toxic heavy metal such as osmium tetroxide (see Rowley 1959; Zavialova et al. 2018 versus this study; Table II). Also, sufficient contrast between pollen wall layers can be achieved by staining sections at the final stage of preparation. Potassium permanganate is used as alternative fixative to osmium tetroxide (Doyle et al. 1975), but can also be used as staining agent for sections (see this study; Table II; Figure 4). The benefit of skipping prefixation and postfixation is that dehydration becomes superfluous and can be reduced to a short cleaning in acetone for 1 min (see previous studies versus this study; Table II). Infiltration is also important for fossil pollen to exclude the entrapment of air within cavities prior to embedding (see previous studies versus Zetter et al. 2002; Zavialova et al. 2018 and this study; Table II). Extraction of the acetone is not needed because of the small sample (single grains) and embedding mould size (see Zetter et al. 2002 versus recent studies; Table II), and evaporation time of acetone can be reduced.

The new protocol presented herein, allows for the pollen grain to be placed directly within the final embedding mould, skipping many of the commonly used steps of fixation and embedding (see previous studies versus this study; Table II). Also, the polymerisation time is reduced due to the small size of the embedding mould (this study; Table II).

One major benefit of using the advanced method presented herein is the time and effort saved during trimming. Since the pollen is already ideally placed within the rather small embedding mould (at the bottom; Figure 2F) finding the pollen grain in the polymerised block is not a problem anymore. Also, by avoiding to trim the upper surface of the specimen block to reach the section plane of the pollen grain, the specimen block can be used directly for ultra-thin sectioning. Therefore, it is not necessary to cut out the object and to re-embed and polymerise it again before cutting the final specimen block. Additionally, because of the flat/smooth inner surface of the embedding mould (microcentrifuge tube lid), the frontal plane of the pyramid is flat enough for direct ultra-thin sectioning (this study; Table II). Previous studies, using standard embedding moulds or the ‘flat embedding’ method (Zetter et al. 2002), struggled finding individual pollen grains within the specimen block. Also, if the pollen grain was positioned close to the wall or far away from the base of the specimen block, direct trimming was excluded and re-embedding enforced (e.g. Zavialova et al. 2018, pp. 141–142).

The commonly used staining agent, uranyl acetate/lead citrate, effectively stains the pollen wall very dense (dark), but it does not fully differentiate the individual layers of the wall (Figure 4). For this conventional staining method (e.g. Hayat 2000), sections made from osmified material are stained for 30 to 45 min in uranyl acetate solution, followed by lead citrate staining for 1 to 5 min, and thoroughly washed in a row of water drops after each staining. The result of this staining method may be that the ektexine and endexine differ in their electron opaqueness. Usually, the ektexine stains electron dense, producing a distinctive contrast, and is therefore often well differentiated from the endexine (less or more electron dense). Still, to differentiate the two exine layers can be problematic, especially if the endexine is thin or discontinuous (Weber & Ulrich 2010). The use of different staining agents on fossil pollen, presented herein (Figure 4), demonstrates that the staining behaviour of acetolysed and/or fossil pollen differs from that of living pollen, due to changes in sporopollenin chemistry (e.g. Yule et al. 2000; Fraser et al. 2014; Zavialova et al. 2018). Experiments with acetolysed recent pollen showed remarkable differences in distinguishing layers of the pollen wall, depending on the staining agent used (Figure 4A, B). Applying KMnO_4_ made the exine layers become clearly visible. For example, the endexine stains electron dense and is differentiated from the ektexine. Even fine structures, like channels or lamination, that are obscured using uranyl acetate/lead citrate, can be observed (Weber & Ulrich 2010; Figure 4A; Table II). In case of the *in situ* fossil pollen grain from the middle Eocene of Germany), the staining results proved to be quite similar (Figure 4C, D). Still, the endexine stains more intensively using KMnO_4_ (Figure 4C), making fine structures clearly visible, compared to staining using the conventional agent (Figure 4D). Interestingly, the contrast in unstained sections of the fossil *Sclersperma* pollen (Figure 3F) is comparable to sections stained with uranyl acetate/lead citrate (Figure 4F). Layering of the exine is difficult to observe in both cases. Staining using KMnO_4_ (Figure 4E) clearly differentiates the layers of the exine compared to conventional staining (Figure 4F). In this case it seems more likely that the thick basal layer is actually a thin compact-continuous foot layer accompanied by a thick continuous-compact endexine (Figure 4E) (see also Harley & Dransfield 2003). Still, as pollen/spore taxa may react differently to chemical treatment, the use of diverse preparation and staining methods is always recommended.

## Conclusion and outlook

The hope is that future studies on fossil pollen will increasingly include combined studies using LM, SEM and TEM. The ultrastructure of pollen usually includes important features that can be used to taxonomically place pollen and make assumptions on evolutionary trends within plant groups/lineages. Unfortunately, in palaeopalynology, TEM studies have been neglected and only a few researchers (mostly in the United States and Russia) have made the effort to document the ultrastructure of fossil pollen/spores. The protocol presented herein will hopefully encourage other palynologists working on fossil pollen to incorporate TEM studies in their research. At least the advanced protocol is fast and easy to follow.
